# Impacts of Hyperbaric Oxygen Therapy (HBOT) on Verbal Scores in Children With Autism: A Secondary Analysis of the HBOT Trial Using Multivariate Analysis of Variance (MANOVA)

**DOI:** 10.7759/cureus.69421

**Published:** 2024-09-14

**Authors:** Tami Peterson, Jessica Dodson, Sheila Burgin, Robert Sherwin, Frederick Strale,

**Affiliations:** 1 Hyperbaric Oxygen Therapy, The Oxford Center, Brighton, USA; 2 Applied Behavior Analysis, The Oxford Center, Brighton, USA; 3 Hyperbaric Oxygen Therapy, Wayne State University School of Medicine, Detroit, USA; 4 Biostatistics, The Oxford Center, Brighton, USA

**Keywords:** ablls, applied behavior analysis (aba), autism spectrum disorder (asd), hyperbaric oxygen therapy (hbot), multivariate analysis of variance (manova), vb-mapp, verbal behavior milestones

## Abstract

Introduction

A secondary analysis employing advanced statistical methodologies constitutes a robust means of validating initial findings in systematic empiricism. The current research will undertake a secondary analysis of the impacts of Hyperbaric Oxygen Therapy (HBOT) on verbal behaviors in children with autism using the original dataset. This approach aims to enhance the robustness of the initial results, thereby providing a deeper understanding of the data and potentially uncovering additional insights.

Materials and methods

From January 2018 to July 2021, all cohorts of autistic children (n = 65) were scheduled, evaluated, and treated at The Oxford Center (TOC) in Brighton and Troy, Michigan, USA. Trained research assistants retrospectively extracted pretest and posttest data from electronic medical records from the Verbal Behavior Milestones Assessment and Placement Program (VB-MAPP) and the Assessment of Basic Language and Learning Skills (ABLLS). This data collection focused on children with autism who received either non-HBOT control with Applied Behavior Analysis (ABA) treatment only or ABA + HBOT interventions.

For the VB-MAPP, the experimental group (ABA + HBOT) included 23 children, while the control group (ABA only) included 12 children. For the ABLLS, the experimental group (ABA + HBOT) consisted of nine children, compared to 21 children in the control group (ABA only). Demographic information was systematically summarized. Two independent sample t-tests were recomputed from the original study. Multivariate Analysis of Variance (MANOVA) were conducted, followed by one-way Analyses of Variance (ANOVA) post hoc analyses to elucidate the findings.

Results

The ages in both groups ranged from 2 to 17 years (M = 5.7 years ± 3.08), with median ages of four years for the experimental group and five years for the control group. The p-values and effect sizes indicated that the two independent sample t-tests from the original study and the MANOVAs from the current research are in agreement. This concordance provided confirmatory evidence for the validity of the pretest and posttest differences in VB-MAPP and ABLLS scores for the control group (ABA only) and the experimental group (ABA + HBOT), highlighting the impact of HBOT on verbal scores in children with autism.

Conclusions

The results from the two independent sample t-tests from the initial study exhibited high alignment with those derived from the current study's MANOVAs. Both statistical methodologies were applied to the same VB-MAPP and ABLLS datasets. The convergence of results from these two distinct statistical analyses may reinforce the credibility of the original research findings. It supports the hypothesis that the combined ABA and HBOT intervention may offer additional benefits over ABA therapy alone, with verbal milestone behaviors in children with autism.

## Introduction

Autism spectrum disorder (ASD) is a neurodevelopmental condition characterized by challenges in verbal and social communication, repetitive behaviors, limited environmental interaction, and literacy difficulties. Individuals with ASD often experience impairments in spoken language. According to the Diagnostic and Statistical Manual of Mental Disorders, Fifth Edition (DSM-5), diagnostic criteria for autism include deficits in social and verbal communication, reduced interactions across various settings, and restrictive or repetitive behaviors that emerge early in development. These symptoms significantly impact social and occupational functioning and are not better explained by other conditions [1,2].

ASD affects individuals across all socioeconomic, racial, and ethnic backgrounds, though it is significantly more common in boys than girls. It profoundly impacts the lives of those affected and their families. In approximately one-third of cases, children may experience a regression in developmental milestones early in life [1,3]. According to the Centers for Disease Control and Prevention (CDC), the prevalence of ASD in children in the United States is currently 1 in 36, and, worldwide, 1 in 100 [1,4].

The current hypothesis for ASD suggests that both genetic predisposition and environmental factors contribute to its development, potentially involving up to 1,000 genes [1,5,6]. The pathophysiology of ASD is complex and includes factors such as oxidative stress, cerebral hypoperfusion, inflammation, mitochondrial dysfunction, and immune dysregulation [1,6-9]. Studies have identified cerebral hypoperfusion, oxidative stress, and neuroinflammation as potential targets for the benefits of Hyperbaric Oxygen Therapy (HBOT) in ASD patients [1,9-12].

Children with autism may struggle with speech and language, making expressing themselves and understanding others difficult. While some may have strong vocabulary skills and engage in detailed conversations, they often face challenges in social situations, such as initiating conversations or making requests [1,13]. Additionally, they may exhibit difficulties with facial expressions, eye contact, body language, and echolalia (repeating others' words). Literacy impairments, such as reading, writing, and comprehension, are also common. Sensory sensitivities to sound can further impact their verbal abilities [1,13].

ASD presents various treatment options, including behavioral, developmental, educational, social-relational, pharmacologic, psychological, and complementary or alternative interventions. Despite early diagnosis and intensive therapy, individuals with ASD often continue to experience significant challenges in social interaction, communication, academic performance, and overall quality of life [1,11]. Consequently, many parents seek additional therapeutic options, such as melatonin, secretin therapy, dietary modifications, vitamin supplements, and HBOT [1,11].

HBOT involves administering 100% oxygen at pressures above atmospheric levels and has demonstrated effects on several physiological processes, including reducing inflammation, improving mitochondrial function, correcting tissue hypoxia, and enhancing the body’s ability to manage oxidative stress. These benefits align with several theories regarding the pathophysiological basis of ASD [1,14].

Reviews by the Cochrane Database and the Agency for Healthcare Research and Quality (AHRQ) have not provided definitive conclusions about HBOT's impact on verbal behavior in individuals with ASD. Many studies either employ interventions that do not adhere to the Undersea and Hyperbaric Medical Society (UHMS) criteria for HBOT, or suffer from inadequate control groups or flawed methodologies.

Systematic reviews and literature on HBOT for ASD report mixed findings, ranging from no benefit to promising outcomes. A significant challenge in synthesizing this body of research is its heterogeneity, stemming from variations in defined outcomes, patient populations, comparator groups (e.g., sham treatments vs. controls), and the specific pressures and oxygen levels used in treatment [1,12].

Despite the existing literature on HBOT's efficacy in treating ASD, there remains a need for further research due to the scarcity of well-conducted trials [1,14-16]. This study is particularly novel and distinct, as it directly examines HBOT's effects on verbal behavior using a secondary analysis with the data presented by Peterson et al., addressing a notable gap in the current research [1].

Study objective

This study will conduct a secondary analysis of Peterson et al.'s [1] original research, which found statistically significant effects of HBOT on improving verbal behaviors among autistic children. The Verbal Behavior Milestones Assessment and Placement Program (VB-MAPP) and the Assessment of Basic Language and Learning Skills (ABLLS) were used in the original analysis.

While the original study used two independent sample t-tests to assess verbal milestones individually, this secondary analysis will employ Multivariate Analysis of Variance (MANOVA) to examine multiple dependent variables within VB-MAPP and ABLLS simultaneously. By doing so, MANOVA aims to enhance the robustness of the findings by accounting for interrelationships among variables and reducing the risk of Type I errors. Consistent results through MANOVA may further validate and strengthen the reliability of the initial findings.

## Materials and methods

Study context and subjects

Between January 2018 and July 2021, a cohort of 65 autistic children was scheduled, treated, and monitored at The Oxford Center (TOC) in Brighton and Troy, Michigan, USA. These outpatient facilities offer a range of clinical services for various conditions, including ASD. The services provided encompass Applied Behavior Analysis (ABA) therapy, nutrition therapy, neurofeedback, music therapy, educational support, HBOT, physical, occupational, and speech therapy. The children at TOC had access to any of these therapies, with HBOT being an optional inclusion based on parental preference [1].

Data acquisition

The study data were retrospectively retrieved from electronic medical records by trained research assistants, to obtain information on ABA child cohort patients who received either non-HBOT (control-ABA only) or HBOT (ABA + HBOT) interventions. Three independent Board-Certified Behavior Analysts (BCBAs), who were not part of the research team, collected the original pretest and posttest VB-MAPP and ABLLS data for both the control and experimental groups. The manuscript adhered to the Consolidated Standards of Reporting Trials (CONSORT) and Strengthening the Reporting of Observational Studies in Epidemiology (STROBE) guidelines [1].

Records of child cohorts aged 2 to 17 years, diagnosed with ASD, were screened for study inclusion. Each child, whether they received HBOT or not, completed a minimum of 40 sessions at 2.0 atmospheres absolute (ATA) during the study period and underwent the VB-MAPP and ABLLS verbal assessments. Children diagnosed with seizure disorders, or genetic or mitochondrial mutations, were excluded from the study. Those who received HBOT were placed in the treatment group, while those who did not were assigned to the control group [1]. Matched pretest-posttest pairs were created to ensure the children’s cohorts differed only by the HBOT intervention. Five males were excluded from the HBOT group due to various reasons: the initial verbal test was administered after the first HBOT session (n = 3), the post-test was administered before the last HBOT session (n = 1), and missing verbal test data (n = 1). In the non-HBOT control group, three males and three females were excluded for not meeting inclusion criteria due to incomplete assessments (n = 2), duplicate entries (n = 1), and missing data (n = 3). The children’s cohorts served as their own controls in the pretest-posttest comparisons, thereby reducing potential bias [1].

HBOT administration

The HBOT sessions were conducted in a Class B chamber approved by the Food and Drug Administration (FDA). This chamber can administer treatments at pressures ranging from 1 to 3 ATA, with an average oxygen concentration of 99.803%, as verified by third-party gas analysis. The specific model used in this trial was the Sechrist 3300H monoplace hyperbaric chamber (Sechrist Industries, Inc., Anaheim, CA, USA). Patients in the HBOT group (n = 32) were exposed to medical-grade oxygen at 2.0 ATA, with pressure increased at 1-2 psi per minute during five sessions per week. During each session, subjects inhaled the oxygen-enriched environment at the treatment depth for 60 minutes without interruption, while being monitored for adverse events (AEs) by a trained hyperbaric technician. The chamber was then depressurized at 1-2 psi per minute back to 1.0 ATA [1].

AEs

AEs were recorded exclusively for subjects in the treatment group in relation to HBOT. All reported AE terms were coded using the Medical Dictionary for Regulatory Activities (MedDRAs) and categorized by System Organ Class (SOC) and Preferred Term (PT). AE data collection began at the start of the study and continued through its conclusion. AEs were summarized by the total number and percentage of subjects who experienced at least one AE. When an AE occurred, it was reported by the hyperbaric technician to the attending nurse and supervising physician.

ABA procedures

All patients received ABA treatment, which was a control variable held constant across groups. Patients in both the control (ABA only) and experimental (ABA + HBOT) groups received the same number of ABA sessions, with a minimum of 25 treatment hours per week. ABA is a one-on-one therapy that utilizes discrete trial training, and mass trials in a naturalistic environment to develop skills that enhance clients' success in their homes, schools, and communities.

Discrete trial training is an ABA modality that simplifies complexity by taking large, gross tasks, reducing them to small, individualized tasks, and teaching them with straightforward and systematic methods. Mass trials are a method within discrete trial training that includes repeatedly presenting the same stimulus until the learner responds correctly. Naturalistic environment training (NET) is a form of ABA that teaches behavioral skills within the natural context of a learning environment. The respective learner’s individual preferences and partialness serve as the motivation [17,18-25].

The effects of a blend of discrete trial training, mass trials, and NET in autistic children are noteworthy, as they can assist with various aspects of learner cognitive, language, social, and adaptive skills development. The benefits of discrete trial training include helping autistic children learn appropriate responses to different situations, which can enhance communication, their relationships with family, classmates, and peers, and overall quality of life. Acquiring skills such as matching, discrimination, and imitation using this form of ABA can enhance learning, which is challenging to obtain in naturalistic settings [17,18-25].

Mass trials assist autistic children with acquiring new behaviors more quickly and efficiently, as exposure to the same or similar stimulus increases. This ABA method can help increase and retain learned behaviors over time by strengthening memory and improving recall abilities. NET assists autistic children with generalization skills transferred from discrete trial training to different contexts, including people, materials, and settings. NET also helps with increased motivation, spontaneity, and engagement by utilizing reinforcements that occur naturally and are aligned with learner interests [17,18-25].

Dependent measures

A BCBA conducted the initial assessment using either the VB-MAPP or ABLLS, depending on the child’s developmental level. Initial goals were established and subsequently reviewed after an observation period. The VB-MAPP and ABLLS assessments were administered to both the control (ABA only) and experimental (ABA + HBOT) groups at baseline (pretest) and again following the 40th HBOT session. Each HBOT session lasted 60 minutes at 2.0 ATA, with at least 40 sessions completed. To control for potential confounding variables, a matched-pair (pretest-posttest) design was employed, with each child serving as their own control. Any changes in medications, and the initiation or discontinuation of other therapies (e.g., speech, physical, and occupational therapy), were documented [1].

VBMAPP

The child cohorts from both the (ABA + HBOT) treatment group (n = 23) and the (ABA only) control group (n = 12) were systematically evaluated by a BCBA at both pretest and posttest stages. The evaluation focused on a broad range of behavioral milestone domains essential for the acquisition of language and social skills. These domains included mand (requesting), tact (labeling), listener skills, visual and perceptual skills, independent play, social interaction, motor imitation, echoic responses (vocal imitation), listener responding, intraverbal skills (conversation), group behavior, and linguistic structure.

Each child was observed during the evaluation, with specific prompts provided by the BCBA to elicit responses related to these domains. The children's performances were rated on a five-point Likert scale, where higher scores reflected better progress across the subscales. The comprehensive assessment aimed to capture the developmental progress of each child cohort in key areas, that are fundamental to communication and social interaction.

The VB-MAPP demonstrated strong internal consistency for this sample. The reliability of the VB-MAPP was evidenced by a Cronbach's alpha of r = 0.936, indicating a high degree of dependability in measuring the intended behavioral domains [1,13]. This reliability reinforces the reliability of the findings, highlighting the effectiveness of the evaluation process in tracking developmental progress.

ABLLS

The child cohorts (n = 30) in both the (ABA + HBOT) treatment group (n = 9) and the (ABA only) control group (n = 21) were assessed by a BCBA at both the pretest and posttest stages. The assessment involved evaluating each child across various behavioral milestone subscales, which included receptive language, requesting (mand), labeling (tact), intraverbals, spontaneous vocalizations, syntax and grammar, social interaction, and generalized responding. These subscales are critical components of the ABLLS, designed to measure key areas of language development, communication, and social functioning in children. Each child's performance was documented on a grid, with ratings reflecting the child's progress on each milestone [16]. The internal consistency of the ABLLS for this sample was robust, as indicated by a Cronbach's alpha of r = 0.869, suggesting a high level of reliability in the assessment tool for this cohort [1,15].

Power analysis

A retrospective power analysis was performed using G*Power 3.1 software (Heinrich-Heine-Universität Düsseldorf, Düsseldorf, Germany) to determine the appropriate sample size needed to detect a significant difference in mean outcomes between the HBOT and non-HBOT groups. The analysis revealed that a total sample size of n = 64 participants would be necessary to achieve a large effect size (ES = 0.80) in the comparison of means between the two groups, with a nominal alpha (α) level of 0.05, using a two-tailed, two independent sample t-test. The power of the test, calculated as 0.882, indicates a high probability of correctly rejecting the null hypothesis if a true difference exists between the groups [1,26].

Given that the current study involved n = 65 participants, it meets the sample size requirements suggested by the power analysis. This suggests that the study is sufficiently powered to detect a meaningful effect, reducing the risk of a Type II error (failing to detect a true effect). Additionally, it implies that the sample size used in this retrospective trial is appropriate for the statistical analysis being conducted, ensuring that the findings are robust and reliable. Notably, no external studies were referenced to estimate the required sample size, as the power analysis was solely based on the parameters relevant to this specific trial [1,26].

Statistical procedures

All descriptive and inferential statistical analyses were conducted using IBM SPSS Statistics for Windows, Version 29 (Released 2022; IBM Corp., Armonk, NY, USA) [27]. The demographic and baseline characteristics of all participants were summarized both overall and by treatment group. For continuous variables, such as age, duration of HBOT treatment in months, the time span from the baseline verbal test to the post-baseline verbal test, the duration from the baseline verbal test to the start of HBOT treatment, the time from the final HBOT session to the post-baseline verbal test, age at VB-MAPP assessment, and age at ABLLS assessment, summary statistics were generated. These included the number of subjects, mean, standard deviation, median, and range.

Categorical variables, such as race/ethnicity and autism severity level, were presented as the number and percentage of subjects within each category. The analysis also focused on summarizing the changes from baseline in VB-MAPP and ABLLS scores, as well as the incidence of adverse and serious events by treatment group. Detailed summaries of all components of the verbal tests were provided, with descriptive statistics calculated for each subject and by study treatment.

To assess the equivalence of the control (ABA only) and experimental (ABA + HBOT) groups, the Chi-square test of independence was employed. This statistical method evaluates whether there is a significant association between categorical variables, thereby determining if the distribution of characteristics is independent across the groups. The resulting p-values from this test will be reported to indicate the statistical significance of any observed differences. These p-values will provide insight into whether any disparities between the control and experimental groups are likely due to random chance or represent a meaningful divergence.

To compare the mean change in overall verbal test scores between the (ABA + HBOT) treatment group and the control (ABA only) group, a two-tailed, two-independent sample t-test will be conducted. An alpha (α) level will be set at 0.05. Statistical significance will be determined for p-values less than 0.05.

Secondary Analysis

Given that this research constitutes a secondary analysis, the pre-existing dataset from the original investigation, which employed a two-independent sample t-test to examine statistically significant differences between the control and experimental groups, specifically focusing on the efficacy of HBOT in enhancing verbal behaviors in children with autism, will be subjected to a more sophisticated reanalysis. This reanalysis will utilize MANOVA, a robust and advanced inferential statistical technique designed to assess the impact, in this case, of one factor with two levels (ABA only - control group and ABA + HBOT - experimental group) on multiple VB-MAPP and ABLLS dependent variables simultaneously. Post hoc one-way Analyses of Variance (ANOVA) will be conducted after the MANOVAs on all VB-MAPP and ABLLS scales to determine specific scale significance and non-significance between the control (ABA only) and experimental (ABA + HBOT) groups.

By employing MANOVA, this secondary analysis seeks to provide a more comprehensive understanding of the underlying relationships between variables, potentially revealing multivariate effects that the initial two-independent sample t-test may have overlooked. This approach not only enhances the rigor of the statistical examination but also offers a more nuanced and multidimensional perspective on the efficacy of HBOT in this specific population. All statistical findings will be presented through both textual descriptions and tables.

Independent ethics committee

This research study was conducted retrospectively, utilizing data obtained through a chart review originally gathered for clinical purposes. The study protocol was submitted to the WIRB-Copernicus Group (WCG® IRB) for ethical review, where it received an exemption (1-1435713-1). The authors hereby affirm that the analysis was conducted in strict adherence to the ethical guidelines established by the 1964 Declaration of Helsinki, including its subsequent amendments and any other comparable ethical standards [28]. These guidelines are foundational in ensuring the protection of human subjects in research, emphasizing the principles of beneficence, justice, and respect for individuals. Furthermore, it should be noted that, subsequent to acquiring the ClinicalTrials.gov Identifier: NCT06043284, the Oxford Recovery Center (ORC), the institution involved in the study, has undergone a name change and is now known as TOC. The study was also registered under another study identification number: OxRS-01-2021. This transition reflects an ongoing commitment to excellence in research and clinical care under the TOC designation, ensuring the continuity of ethical standards and scientific rigor across its clinical investigations.

## Results

Table 1 below presents the crosstabulation of the 65 child cohorts enrolled in the original retrospective trial [1]. Two-way Chi-squared indicated (Chi-squared = 8.244, p = 0.004, df = 1). There is a significant association between the group (ABA + HBOT experimental vs. ABA control) and the assessment used (VB-MAPP or ABLLS). Specifically, in the ABA + HBOT experimental group, more participants (23 out of 32) were assessed using VB-MAPP compared to ABLLS (9 out of 32). In the ABA control group, more participants (21 out of 33) were evaluated using ABLLS compared to VB-MAPP (12 out of 33). The significant Chi-square result suggests that the type of assessment used (VB-MAPP or ABLLS) is not independent of the group assignment. This implies that ABA + HBOT experimental group participants were more likely to be assessed with VB-MAPP, while those in the ABA control group were more likely to be evaluated with ABLLS.

**Table 1:** VB-MAPP and ABLLS crosstabulation VB-MAPP: Verbal Behavior Milestones Assessment and Placement Program; ABLLS: Assessment of Basic Language and Learning Skills-Revised; ABA: Applied Behavior Analysis; HBOT: Hyperbaric Oxygen Therapy

Group	VB-MAPP	ABLLS	(VB-MAPP + ABLLS)
ABA + HBOT (experimental)	23	9	32
ABA (control)	12	21	33
Experimental + control	35	30	65

Thirty-two child cohorts were assigned to the experimental group (ABA + HBOT), comprising 31 males and 1 female (Tables 1-2). The remaining 33 child cohorts were allocated to the control group (ABA only), which did not receive HBOT and consisted of 27 males and 6 females. The baseline characteristics across the two groups were comparable (Table 2), as indicated by non-significant two-way Chi-squares and two independent sample t-tests (p-values, p > 0.05), demonstrating statistical equality between the experimental (ABA + HBOT) and control (ABA only) groups. These p-values are detailed in Table 2, underscoring the lack of statistically significant differences in key demographic and baseline variables between the groups before the intervention [1].

**Table 2:** Baseline characteristics of the experimental (ABA + HBOT) and control (ABA only) groups VB-MAPP: Verbal Behavior Milestones Assessment and Placement Program; ABLLS: Assessment of Basic Language and Learning Skills-Revised; ABA: Applied Behavior Analysis; HBOT: Hyperbaric Oxygen Therapy

Variable	Statistic	Experimental (ABA + HBOT) (n = 32)	Control (ABA only) (n = 33)	Total (n = 65)	p-value
Treatment months for HBOT	n	32	0	32	N/A
	Mean	5.53		5.53	
	SD	1.08		1.08	
	Median	6		6	
	Minimum	2		2	
	Maximum	6		6	
Months from baseline verbal test to post baseline verbal test	n	32	33	65	0.215
	Mean	5.53	5.8	5.7	
	SD	1.08	0.73	0.8	
	Median	6	6	6	
	Minimum	2	4	2	
	Maximum	6	8	8	
Months from baseline verbal test to first HBOT treatment	n	32	0	32	N/A
	Mean	1		1	
	SD	1.22		1.22	
	Median	0		0	
	Minimum	0		0	
	Maximum	4		4	
Months from last HBOT treatment to post baseline verbal test	n	32	0	32	N/A
	Mean	2.81		2.81	
	SD	1.4		1.4	
	Median	3		3	
	Minimum	0		0	
	Maximum	5		5	
Age, years	n	32	33	65	0.084
	Mean	5.1	6.55	5.7	
	SD	2.93	3.58	3.08	
	Median	4	5	5	
	Minimum	2	2	2	
	Maximum	17	16	17	
Age, (VB-MAPP) years	n	23	12	35	0.744
	Mean	3.96	4.08	4	
	SD	1.07	1.08	3.08	
	Median	4	5	5	
	Minimum	2	2	2	
	Maximum	17	16	17	
Age, (ABLLS) years	n	9	21	30	0.921
	Mean	8.1	7.96	7.7	
	SD	4.01	3.76	3.46	
	Median	8	7	7	
	Minimum	3	4	3	
	Maximum	17	16	17	
Race/ethnicity, n (%)	African American Indian	1 (3.1%)	0 (0.0%)	1 (1.5%)	0.67
	Asian	0 (0.0%)	1 (3.0%)	1 (1.5%)	
	Hispanic	1 (3.1%)	0 (0.0%)	1 (1.5%)	
	Middle Eastern	4 (12.5%)	3 (9.1%)	7 (10.8%)	
	White	3 (9.4%)	2 (6.1%)	5 (7.7%)	
	American Indian	18 (56.3%)	23 (69.7%)	41 (63.1%)	
	Unspecified	0 (0.00%)	1 (3.0%)	1 (1.5%)	
		5 (15.6%)	4 (12.1%)	9 (13.8)	
Autism severity level, n (%)	1	1 (3.1%)	3 (9.1%)	4 (6.2%)	0.495
	2	9 (28.1%)	11 (33.3%)	20 (30.8%)	
	3	22 (68.8%)	19 (57.6%)	41 (63.1%)	
Gender (%)	Male	31 (96.9%)	27 (81.8%)	89.20%	0.103
	Female	1 (3.1%)	6 (8.2%)	10.80%	

The average age of the children across both groups was 5.7 years (M = 5.7 ± 3.08), ranging from 2 to 17 years. This broad age range reflects the study's inclusion of a diverse sample of participants, encompassing early childhood through adolescence. The statistical non-significance of the baseline characteristics is crucial in establishing the validity of the study’s findings, as it ensures that any observed differences in outcomes between the experimental (ABA + HBOT) and control (ABA only) groups are not attributable to pre-existing disparities, but rather to the intervention itself. The balanced distribution of vital demographic factors may further enhance the trial's internal validity, allowing for a more rigorous examination of the effects of HBOT on the study population [1].

Over 63% of the pediatric cohort exhibited an autism severity level of 3, indicating profound impairment. In comparison, approximately 31% were classified with severity level 2, reflecting moderate impairment, while a minority of over 6% were categorized at severity level 1, denoting a milder form of autism [1].

Within the control group (ABA only), assessments included the VB-MAPP administered to 12 participants and the ABLLS administered to 21 participants. For those in the experimental (ABA + HBOT) group, the intervention was conducted over an average duration of 5.53 months (M ± 1.08), with the range extending from two to six months [1].

Age-appropriate verbal assessments were carried out, with 35 children (M = 5.7 years ± 3.08) undergoing the VB-MAPP (ages 2 to 17 years) and 30 children (M = 7.7 years ± 3.46) undergoing the ABLLS (ages 3 to 17 years). Within the experimental group (ABA + HBOT), 23 children (M = 3.96 years ± 1.07) were administered the VB-MAPP, while nine children (M = 8.1 years ± 4.01) received the ABLLS [1].

VB-MAPP two independent sample t-tests from Peterson et al.'s study

The evaluation of participants using the VB-MAPP individual scales (Table 3) revealed substantial mean differences, with large effect sizes ranging from -0.743 to -1.650, and a total score effect size of -1.23. Statistically significant differences (p < 0.05) were observed between difference scores across both the control (ABA only) and experimental (ABA + HBOT) groups, underscoring the impact of the HBOT intervention relative to the control condition [1].

**Table 3:** VB-MAPP change in difference scores between the control (ABA) and experimental (ABA + HBOT) groups Cohen's d: Small effect size = 0.20; Medium effect size = 0.50; Large effect size = 0.80 or higher ABA: Applied Behavior Analysis; HBOT: Hyperbaric Oxygen Therapy; VB-MAPP: Verbal Behavior Milestones Assessment and Placement Program

Scale	Time	HBOT mean (SD), n = 24	Non-HBOT mean (SD), n = 13	Mean difference	95% CI	SE	Effect size (Cohen's d)	95% CI	p-value
Mand	Pretest	2.41 (3.34)	2.96 (2.85)						
	Posttest	6.43 (4.00)	4.33 (2.82)						
	Difference	4.02 (2.37)	1.38 (0.83)	2.65	1.53, 3.77	0.55	-1.33	-2.09, -0.553	<0.0001
Tact	Pretest	2.24 (3.02)	3.00 (2.88)						
	Posttest	6.50 (4.11)	4.17 (3.03)						
	Difference	4.26 (2.96)	1.17 (1.05)	3.09	1.69, 4.50	0.69	-1.24	-1.99, -0.474	<0.0001
Listener responding (LR)	Pretest	3.20 (3.10)	3.92 (3.64)						
	Posttest	7.50 (4.03)	5.71 (3.31)						
	Difference	4.30 (2.42)	1.79 (1.41)	2.51	0.96, 4.06	0.76	-1.18	-1.92, -0.416	0.0023
Visual perceptual skills and matching-to-sample (VP-MTS)	Pretest	4.57 (2.77)	5.38 (3.49)						
	Posttest	7.98 (3.65)	6.79 (3.12)						
	Difference	3.41 (1.86)	1.42 (1.13)	2	0.80, 3.19	0.59	-1.21	-1.96, -0.444	0.0018
Independent play	Pretest	4.22 (3.92)	5.63 (2.82)						
	Posttest	8.26 (4.10)	6.96 (2.56)						
	Difference	4.04 (2.75)	1.33 (1.37)	2.71	1.29, 4.13	0.70	-1.14	-1.88, -0.382	0.0005
Social play	Pretest	2.22 (2.27)	2.88 (2.14)						
	Posttest	6.22 (2.68)	4.50 (2.29)						
	Difference	4.00 (1.97)	1.63 (1.19)	2.38	1.11, 3.64	0.62	-1.36	-2.12, -0.581	0.0006
Motor imitation	Pretest	1.93 (2.44)	2.29 (2.21)						
	Posttest	6.24 (2.93)	4.00 (2.44)						
	Difference	4.30 (2.44)	1.71 (1.27)	2.6	1.32, 3.87	0.63	-1.22	-1.97, -0.458	0.0002
Echoic	Pretest	1.65 (2.34)	4.08 (4.25)						
	Posttest	5.98 (3.40)	4.29 (4.16)						
	Difference	4.33 (3.04)	0.21 (0.33)	4.12	2.79, 5.45	0.64	-1.65	-2.46, -0.840	<0.0001
Spontaneous vocalization	Pretest	1.93 (1.73)	2.42 (1.74)						
	Posttest	4.96 (2.50)	3.13 (1.57)						
	Difference	3.02 (2.28)	0.71 (0.78)	2.31	1.24, 3.39	0.53	-1.21	-1.96, 0.445	0.0001
Listener responding by function, feature, and class (LRFFC)	Pretest	0.57 (1.23)	0.67 (2.02)						
	Posttest	3.28 (2.71)	1.13 (2.07)						
	Difference	2.72 (2.13)	0.46 (0.94)	2.26	1.20, 3.32	0.52	-1.24	-1.99, 0.475	0.0001
Intraverbal	Pretest	0.35 (0.83)	0.58 (1.29)						
	Posttest	2.72 (2.40)	1.46 (1.66)						
	Difference	2.37 (1.86)	0.88 (0.93)	1.49	0.53, 2.46	0.47	-0.928	-1.63, 0.189	0.0033
Group behavior	Pretest	0.89 (1.87)	2.13 (2.25)						
	Posttest	4.67 (2.29)	3.25 (1.75)						
	Difference	3.78 (1.92)	1.13 (1.32)	2.66	1.39, 3.92	0.62	-1.52	-2.30, 0.726	0.0002
Linguistic structure	Pretest	0.74 (1.57)	1.00 (1.71)						
	Posttest	2.59 (2.64)	1.63 (2.43)						
	Difference	1.85 (1.91)	0.63 (0.91)	1.22	0.25, 2.19	0.48	-0.743	-1.46, 0.018	0.0151
Total score	Pretest	26.91 (27.06)	36.92 (27.48)						
	Posttest	73.33 (35.19)	51.33 (26.04)						
	Difference	46.41 (20.14)	14.42 (6.99)	32	22.44, 41.51	4.66	-1.23	-1.91, 0.548	<0.0001

ABLLS two independent sample t-tests from Peterson et al.’s study

For patients assessed using the ABLLS (Table 4), notable mean differences were observed, with effect sizes ranging from small to medium (-0.114 to -0.773) and an overall effect size of 0.487. However, the differences in total scores between the baseline and post-baseline periods were not statistically significant (p = 0.2024) across the treatment groups. This lack of significance is likely due to low statistical power, which results from the high within-group variability inherent in children with autism, as observed between the control and experimental groups, and the small sample size (n = 9) in the ABLLS experimental (ABA + HBOT) group.

**Table 4:** ABLLS change in difference scores between the control (ABA) and experimental (ABA + HBOT) groups Cohen's d: Small effect size = 0.20; Medium effect size = 0.50; Large effect size = 0.80 or higher ABLLS: Assessment of Basic Language and Learning Skills; ABA: Applied Behavior Analysis; HBOT: Hyperbaric Oxygen Therapy

Scale	Time	HBOT mean (SD), n = 24	Non-HBOT mean (SD), n = 13	Mean difference	95% CI	SE	Effect size (Cohen's d)	95% CI for (Cohen's d)	p-value
Receptive language	Pretest	76.56 (37.26)	88.71 (56.12)						
	Posttest	134.78 (47.22)	134.29 (63.15)						
	Difference	58.22 (43.18)	45.57 (33.23)	12.65	-17.00, 42.20	14.47	-0.348	-1.13, 0.441	0.3895
Requests	Pretest	52.22 (24.67)	36..00 (27.47)						
	Posttest	96.22 (36.75)	61.00 (34.39)						
	Difference	44.00 (31.13)	25.00 (21.38)	19.22	-1.05, 39.05	9.79	-0.773	-1.57, 0.039	0.0623
Labeling	Pretest	52.89 (43.49)	49.33 (48.82)						
	Posttest	99.56 (55.07)	86.48 (61.90)						
	Difference	46.67 (43.83)	37.14 (31.46)	9.52	-19.40, 38.44	14.12	-0.269	-1.05, 0.518	0.5055
Intraverbal	Pretest	42.44 (35.21)	33.48 (36.07)						
	Posttest	93.00 (55.10)	67.48 (55.04)						
	Difference	50.56 (40.47)	34.00 (36.71)	15.56	-14.31, 47.42	15.07	0.438	-1.22, 0.355	0.2813
Spontaneous vocalizations	Pretest	23.89 (12.13)	20.71 (13.07)						
	Posttest	30.11 (8.19)	28.00 (10.19)						
	Difference	6.22 (11.68)	7.29 (8.21)	-1.06	8.68, 6.55	3.72	-0.114	-0.668, 0.894	0.7769
Syntax and grammar	Pretest	18.44 (15.83)	12.90 (21.39)						
	Posttest	37.78 (28.87)	24.67 (25.22)						
	Difference	19.33 (16.39)	11.76 (17.92)	7.57	-6.71, 21.85	6.97	-0.433	-1.22, 0.360	0.2867
Social interactions	Pretest	41.22 (26.07)	28.05 (20.33)						
	Posttest	76.33 (40.86)	50.33 (27.71)						
	Difference	35.11 (25.97)	21.00 (22.29)	12.83	-4.24, 29.89	8.33	-0.613	-1.41, 0.189	0.1349
Generalized responding	Pretest	7.78 (7.10)	9.76 (8.61)						
	Posttest	16.56 (9.50)	17.52 (7.90)						
	Difference	8.78 (7.45)	7.76 (6.04)	1.02	-4.27, 6.30	2.58	-0.157	-0.937, 0.626	0.6966
Total score of assessment of language and basic living skills	Pretest	315.44 (154.65)	278.95 (197.74)						
	Posttest	584.33 (238.25)	469.76 (256.28)						
	Difference	268.89 (182.05)	190.81 (135.26)	78.08	-44.43, 200.59	59.81	-0.487	-1.14, 0.369	0.2024

MANOVA results: VB-MAPP with ABA (control) vs. ABA + HBOT (experimental)

As indicated in Table 5, the multivariate tests (Pillai's Trace, Wilks' Lambda, Hotelling's Trace, and Roy's Largest Root) show a significant overall effect of the group variable on the combined dependent variables (VB-MAPP subscale differences). The p-value was equal to 0.037. The effect size (partial η²) for the group effect was 0.596, indicating a strong group effect across all VB-MAPP subscales.

**Table 5:** VB-MAPP Omnibus MANOVA model Partial η²: Small effect size = 0.01; Medium effect size = 0.06; Large effect size = 0.14 or higher MANOVA: Multivariate Analysis of Variance; VB-MAPP: Verbal Behavior Milestones Assessment and Placement Program

MANOVA effect		Value	F	Hypothesis df	Error df	p-value	Partial η²
Intercept	Pillai's Trace	0.834	8.137	13	21	<0.001	0.834
	Wilks' Lambda	0.166	8.137	13	21	<0.001	0.834
	Hotelling's Trace	5.037	8.137	13	21	<0.001	0.834
	Roy's Largest Root	5.037	8.137	13	21	<0.001	0.834
Group	Pillai's Trace	0.596	2.379	13	21	0.037	0.596
	Wilks' Lambda	0.404	2.379	13	21	0.037	0.596
	Hotelling's Trace	1.473	2.379	13	21	0.037	0.596
	Roy's Largest Root	1.473	2.379	13	21	0.037	0.596

VB-MAPP between-subjects effects for post hoc tests (one-way ANOVAs)

Given that the Omnibus MANOVA group effect was significant for multiple VB-MAPP subscales, with the ABA + HBOT (experimental) group consistently outperforming the ABA-only (control) group across various domains, we conducted a series of post hoc one-way ANOVAs, with results as follows: VB-MAPPMandDiff: F(1,33) = 13.937, p < 0.001, partial η² = 0.297; VB-MAPPTactDiff: F(1,33) = 12.147, p = 0.001, partial η² = 0.269; VB-MAPPListenerRespondingDiff (LR): F(1,33) = 10.910, p = 0.002, partial η² = 0.248; VB-MAPPVisualPerceptualSkillsanMatchingtoSampleDiff (VP-MTS): F(1,33) = 11.493, p = 0.002, partial η² = 0.258; VB-MAPPIndependentPlayDiff: F(1,33) = 10.242, p = 0.003, partial η² = 0.237; VB-MAPPSocialPlayDiff: F(1,33) = 14.596, p < 0.001, partial η² = 0.307; VB-MAPPMotorImitationDiff: F(1,33) = 11.802, p = 0.002, partial η² = 0.263; VB-MAPPEchoicDiff: F(1,33) = 21.520, p < 0.001, partial η² = 0.395; VB-MAPPSpontaneousVocalizationDiff: F(1,33) = 11.513, p = 0.002, partial η² = 0.259; VB-MAPPListenerRespondingbyFunction,Feature,andClassDiff (LRFFC): F(1,33) = 12.168, p = 0.001, partial η² = 0.269; VB-MAPPIntraverbalDiff: F(1,33) = 6.785, p = 0.014, partial η² = 0.171; VB-MAPPGroupBehaviorDiff: F(1,33) = 18.293; p < 0.001, partial η² = 0.357; VB-MAPPLinguisticStructureDiff: F(1,33) = 4.358, p = 0.045, partial η² = 0.117; VB-MAPPTotalScoreDiff: F(1,33) = 29.735, p < 0.001, partial η² = 0.474.

The experimental group (ABA + HBOT) significantly outperformed the control group (ABA only) across several VB-MAPP subscales, particularly in echoic, group behavior, and overall total scores, with moderate to large effect sizes observed. These findings suggest that adding HBOT to ABA therapy contributes positively to various verbal behavior and social play metrics in children with developmental challenges, as measured by the VB-MAPP.

The larger partial η² for the total score (0.474) compared to the individual VB-MAPP scales can be attributed to the following reasons: (1) Aggregated variance: the total score encompasses a broader range of skills and behaviors, aggregating the variance from multiple scales. This aggregation often results in a larger effect size because it captures more comprehensive differences between groups. (2) Reduced measurement error: when combining multiple scales into a total score, the measurement error associated with individual scales can be reduced. This reduction in error can lead to a more accurate and larger effect size for the total score. (3) Cumulative impact: the total score reflects the cumulative impact of all the assessed skills and behaviors. Even if individual scales show smaller effect sizes, their combined effect can be more substantial, leading to a larger partial η² for the total score. (4) Statistical power: the total score may have higher statistical power due to the increased sample size and variability it represents. This higher power can detect more significant differences between groups, contributing to a larger effect size.

The total score's partial η² is larger because it integrates the variance and effects from multiple scales, which reduces measurement error and benefits from higher statistical power.

MANOVA results: ABLLS with ABA (control) vs. ABA + HBOT (experimental)

Table 6 below indicates that the multivariate tests (Pillai's Trace, Wilks' Lambda, Hotelling's Trace, and Roy's Largest Root) show a non-significant overall effect of the group variable on the combined ABLLS dependent variables (ABLLS scale differences), with p = 0.162. The overall effect size (partial η²) is large at 0.390.

**Table 6:** ABLLS Omnibus MANOVA model Partial η² is equal to partial eta squared Partial η²: Small effect size = 0.01; Medium effect size = 0.06; Large effect size = 0.14 or higher MANOVA: Multivariate Analyses of Variance; ABLLS: Assessment of Basic Language and Learning Skills

MANOVA effect		Value	F	Hypothesis df	Error df	p-value	Partial η²
Intercept	Pillai's Trace	0.774	9.002	8	21	<0.001	0.774
	Wilks' Lambda	0.226	9.002	8	21	<0.001	0.774
	Hotelling's Trace	3.429	9.002	8	21	<0.001	0.774
	Roy's Largest Root	3.429	9.002	8	21	<0.001	0.774
Group	Pillai's Trace	0.390	1.681	8	21	0.162	0.390
	Wilks' Lambda	0.610	1.681	8	21	0.162	0.390
	Hotelling's Trace	0.640	1.681	8	21	0.162	0.390
	Roy's Largest Root	0.640	1.681	8	21	0.162	0.390

The existence of a non-significant Omnibus MANOVA p-value (p = 0.162) alongside a large (partial η² = 0.390) effect size can be explained by several factors: (1) Sample size: a small sample size can lead to a lack of statistical power, making it difficult to detect significant differences even when the effect size is large. In other words, the study might not have enough participants to achieve statistical significance despite a strong effect size. (2) High variability: if there is high within-group variability, it can obscure the differences between groups. This means that even though the effect size is large, the variability within each group makes it harder to achieve statistical significance. (3) Effect size vs. statistical significance: effect size and statistical significance measure different aspects. The effect size (partial eta squared) indicates the magnitude of the difference between groups, while the p-value indicates whether the observed effect is likely to have occurred by chance. A large effect size suggests a meaningful difference. However, if the sample size is small or variability is high, the p-value might still be non-significant. (4) Type II error: a non-significant p-value could also indicate a Type II error, where the test fails to detect a true effect. This can happen when the study is underpowered, meaning it doesn’t have enough data to detect a significant effect confidently.

The sizeable partial Eta squared indicates a strong effect. However, the non-significant p-value suggests that the study might lack sufficient power or have high variability, which could prevent the detection of statistical significance.

ABLLS between-subjects effects for post hoc tests (one-way ANOVAs)

The results of the post hoc one-way ANOVAs are as follows: ABLLSReceptiveDiff: F(1,28) = 0.764, p = 0.390, partial η² = 0.027; ABLLSRequestsDiff: F(1,28) = 3.769, p = 0.062, partial η² = 0.119; ABLLSLabelingDiff: F(1,28) = 0.455, p = 0.505, partial η² = 0.016; ABLLSIntraverbalsDiff: F(1,28) = 1.207, p = 0.281, partial η² = 0.041; ABLLSSpontaneousVocalizationDiff: F(1,28) = 0.082, p = 0.777, partial η² = 0.003; ABLLSSyntaxGrammarDiff: F(1,28) = 1.179, p = 0.287, partial η² = 0.040; ABLLSSocialInteractionsDiff: F(1,28) = 2.370, p = 0.135, partial η² = 0.078; ABLLSGeneralizedRespondingDiff: F(1,28) = 0.155, p = 0.697, partial η² = 0.006; ABLLSTotalScoreDiff: F(1,28) = 1.704, p = 0.202, partial η² = 0.057.

The p-values ranged from 0.062 to 0.777, indicating no statistical significance. The effect sizes (partial η²) for most subscales were small, with a few moderate, ranging from 0.003 to 0.119. This indicates that group differences explained a limited portion of the variance in ABLLS subscale scores.

The inclusion of HBOT alongside ABA therapy did not produce statistically significant improvements across all ABLLS subscales compared to ABA alone. However, there was a near-significant effect in the requests subscale, suggesting a possible trend that could be explored with a larger sample size.

Two independent sample t-tests from Peterson et al.’s study vs. MANOVA for VB-MAPP and ABLLS from current study

Table 7 below illustrates the alignment of results between the two independent sample t-tests and MANOVA in terms of p-values and effect sizes. This may provide a nuanced understanding of the validity of the original results and the consistency of findings across both statistical approaches.

**Table 7:** Two independent sample t-test vs. MANOVA p-values and effect sizes for VB-MAPP and ABLLS Cohen's d: Small effect size = 0.20; Medium effect size = 0.50; Large effect size = 0.80 or higher Partial η² is equal to partial eta squared Partial η²: Small effect size = 0.01; Medium effect size = 0.06; Large effect size = 0.14 or higher MANOVA: Multivariate Analysis of Variance; VB-MAPP: Verbal Behavior Milestones Assessment and Placement Program; ABLLS: Assessment of Basic Language and Learning Skills-Revised

Dependent variable	Two independent sample t-test (p-value)	Two independent sample t-test effect size (Cohen's d)	MANOVA (p-value)	MANOVA effect size (partial η²)
VB-MAPPMandDiff	<0.001	-1.329	<0.001	0.297
VB-MAPPTactDiff	0.001	-1.241	0.001	0.269
VB-MAPPLRDiff	0.002	-1.176	0.002	0.248
VB-MAPPVPMTSDiff	0.002	1.207	0.002	0.258
VB-MAPPIndependentPlayDiff	0.003	-1.140	0.003	0.237
VB-MAPPSocialPlayDiff	<0.001	-1.361	<0.001	0.307
VB-MAPPMotorImitationDiff	0.002	-1.223	0.002	0.263
VB-MAPPECHOICDiff	<0.001	-1.652	<0.001	0.395
VB-MAPPSpontaneousVocalizationDiff	0.002	-1.208	0.002	0.259
VB-MAPPLRFFDiff	0.001	-1.242	0.001	0.269
VB-MAPPIntraverbalDiff	0.014	-0.928	0.014	0.171
VB-MAPPGroupBehaviorDiff	<0.001	-1.523	<0.001	0.357
VB-MAPPLinguisticStructureDiff	0.045	-0.743	0.045	0.117
VB-MAPPTotalDiff	<0.001	-1.942	<0.001	0.474
ABLLSReceptiveDIFF	0.390	-0.348	0.390	0.027
ABLLSRequestsDIFF	0.062	-0.773	0.062	0.119
ABLLSLabelingDIFF	0.505	-0.269	0.505	0.016
ABLLSIntraverbalDIFF	0.281	-0.438	0.281	0.041
ABLLSSpontaneousVocalizationDIFF	0.777	0.114	0.777	0.003
ABLLSSyntaxGrammarDIFF	0.287	-0.433	0.287	0.040
ABLLSSocialInteractionDIFF	0.135	-0.613	0.135	0.078
ABLLSGeneralizedRespondingDIFF	0.697	-0.157	0.697	0.006
ABLLSTotalDIFF	0.202	-0.520	0.202	0.057

## Discussion

This research conducted a secondary analysis of the original study by Peterson et al., which found significant improvements in verbal behaviors among children with autism through HBOT [1]. The initial analysis used two independent sample t-tests to examine verbal milestone variables. In contrast, this study employed MANOVA to analyze multiple dependent variables simultaneously, offering a more comprehensive statistical approach.

The p-values from the two independent sample t-tests and MANOVA post hoc one-way ANOVAs for VB-MAPP variables consistently aligned, indicating strong statistical significance across both methods. This consistency suggests that both techniques may effectively identify the same variables as significant, with large effect sizes perhaps further reinforcing the robustness of the findings. The largest effect sizes were observed in the VB-MAPP total difference (Cohen's d = -1.942; partial η² = 0.474), indicating a substantial impact.

The alignment of large negative Cohen's d values and large partial η² values highlights the differences observed in the VB-MAPP measures between groups. The strong correspondence in p-values and effect sizes across VB-MAPP domains provides compelling evidence of group differences.

For the ABLLS measures, p-values from both the two independent sample t-tests and MANOVA generally showed non-significant results (p > 0.05), indicating agreement that these measures do not significantly differ between groups. However, the observed mean differences between the control (ABA only) and experimental (ABA + HBOT) groups were noteworthy, with effect sizes ranging from small to moderate, suggesting practical significance despite the lack of statistical significance.

The Omnibus MANOVA model results for ABLLS indicated a large effect size (partial η² = 0.390). However, the non-significant p-values suggest a potential issue with statistical power, likely due to the small sample size (n = 9) in the ABLLS experimental group. Increasing the sample size could improve statistical power, providing a more robust test of the hypotheses and offering deeper insights into the effectiveness of the intervention.

While the observed mean differences in ABLLS are substantial, the non-significant p-values and small to moderate effect sizes warrant cautious interpretation. Enhancing the sample size and conducting more targeted analyses could clarify the intervention's impact.

The alignment between the two independent sample t-tests and MANOVA results, particularly in p-values and effect sizes, underscores the robustness of the VB-MAPP findings, demonstrating significant group differences. In contrast, despite non-significant outcomes, the consistency in ABLLS results suggests a lack of meaningful group differences in these domains, most likely due to insufficient statistical power. This comparison illustrates the effectiveness of MANOVA in confirming and extending findings from more straightforward statistical tests, especially when multivariate considerations are crucial.

Applying MANOVA aimed to enhance the analysis's methodological rigor by accounting for potential interrelationships among variables, thereby reducing the likelihood of Type I errors. The MANOVA findings, as emphasized, were consistent with the original study, reinforcing the reliability and validity of Peterson et al.'s original conclusions [1]. By addressing limitations in the previous analysis, MANOVA provides a more nuanced interpretation of the data, offering deeper insights into HBOT's efficacy in autism intervention. The approach also improves the generalizability of the results, extending their applicability to more diverse contexts.

Limitations

The small sample size, (reduced power) especially in the ABLLS experimental group (n = 9), likely led to non-significant p-values in the ABLLS measures, even though there were substantial mean differences and moderate to large effect sizes. The reduced power may have made it difficult to detect true differences between the control and experimental groups. 

Working with pre-existing data may not suit a MANOVA. This can lead to unmeasured confounding variables or missing relevant factors. The initial study’s design and data collection methods might limit generalizability, especially if there were biases or inconsistencies not addressed in the original analysis.

While MANOVA allows for the analysis of multiple dependent variables at once, it requires certain assumptions, such as the equality of covariance matrices and multivariate normality. If these assumptions are violated, the validity of the results could be compromised.

The generalizability of the findings may be limited by the specific characteristics of the sample, including the severity of autism symptoms, age, and the specific nature of the HBOT intervention used. These factors could influence the applicability of the results to other populations or settings.

The two independent sample t-tests and MANOVA results show consistency in identifying significant variables. This does not rule out the possibility of Type I or Type II errors, especially given the data’s complexity and multiple comparisons. Future studies should replicate these results with alternative statistical approaches or larger datasets to confirm their robustness.

These limitations necessitate a cautious interpretation of the results. Future research should focus on increasing sample sizes, ensuring rigorous data collection methods, and exploring additional variables that may influence the outcomes.

## Conclusions

The results from the two independent sample t-tests exhibit high concordance with those derived from the MANOVA. Both statistical methodologies were applied to the same VB-MAPP and ABLLS datasets. The convergence of results from these two distinct statistical analyses may reinforce the credibility of the original research findings and support the hypothesis that the combined ABA and HBOT intervention may offer additional benefits over ABA therapy alone for verbal milestone behaviors in children with autism.

## References

[1] Peterson T, Sherwin R, Hosey T, Close N, Strale F Jr (2024). The effects of hyperbaric oxygen treatment on verbal scores in children with autism spectrum disorder: a retrospective trial. Cureus.

[2] Murphy CM, Wilson CE, Robertson DM (2016). Autism spectrum disorder in adults: diagnosis, management, and health services development. Neuropsychiatr Dis Treat.

[3] Tonge BJ (2002). Autism, autistic spectrum and the need for better definition. Med J Aust.

[4] (2024). Data and statistics on autism spectrum disorder. https://www.cdc.gov/ncbddd/autism/data.html.

[5] Hallmayer J, Cleveland S, Torres A (2011). Genetic heritability and shared environmental factors among twin pairs with autism. Arch Gen Psychiatry.

[6] De Rubeis S, He X, Goldberg AP (2014). Synaptic, transcriptional and chromatin genes disrupted in autism. Nature.

[7] Doenyas C (2018). Gut microbiota, inflammation, and probiotics on neural development in autism spectrum disorder. Neuroscience.

[8] Bjorklund G, Saad K, Chirumbolo S, Kern JK, Geier DA, Geier MR, Urbina MA (2016). Immune dysfunction and neuroinflammation in autism spectrum disorder. Acta Neurobiol Exp (Wars).

[9] Boddaert N, Zilbovicius M (2002). Functional neuroimaging and childhood autism. Pediatr Radiol.

[10] Rose S, Niyazov DM, Rossignol DA, Goldenthal M, Kahler SG, Frye RE (2018). Clinical and molecular characteristics of mitochondrial dysfunction in autism spectrum disorder. Mol Diagn Ther.

[11] Bjørklund G, Kern JK, Urbina MA (2018). Cerebral hypoperfusion in autism spectrum disorder. Acta Neurobiol Exp.

[12] Podgórska-Bednarz J, Perenc L (2021). Hyperbaric oxygen therapy for children and youth with autism spectrum disorder: a review. Brain Sci.

[13] Sundberg M (2008). VB-MAPP Verbal Behavior Milestones Assessment and Placement Program: A Language and Social Skills Assessment Program for Children With Autism Or Other Developmental Disabilities: Guide. https://www.scirp.org/reference/referencespapers?referenceid=1237325.

[14] Abdel-Rahman EA, Zaky EA, Aboulsaoud M, Elhossiny RM, Youssef WY, Mahmoud AM, Ali SS (2021). Autism spectrum disorder (ASD)-associated mitochondrial deficits are revealed in children's platelets but unimproved by hyperbaric oxygen therapy. Free Radic Res.

[15] Partington J (1998). The Assessment of Basic Language and Learning Skills ABLLS: Scoring Instructions and IEP Development Guide. Behavior Analysts.

[16] Rossignol DA, Frye RE (2012). A review of research trends in physiological abnormalities in autism spectrum disorders: immune dysregulation, inflammation, oxidative stress, mitochondrial dysfunction and environmental toxicant exposures. Mol Psychiatry.

[17] Peterson T, Dodson J, Hisey A, Sherwin R, Strale F (2024). Examining the effects of discrete trials, mass trials, and naturalistic environment training on autistic individuals using repeated measures. Cureus.

[18] Geiger KB, Carr JE, Leblanc LA, Hanney NM, Polick AS, Heinicke MR (2012). Teaching receptive discriminations to children with autism: a comparison of traditional and embedded discrete trial teaching. Behav Anal Pract.

[19] Haq SS, Aranki J (2019). Comparison of traditional and embedded DTT on problem behavior and responding to instructional targets. Behav Anal Pract.

[20] Anwar A, Sutadi R, Miranda C (2019). Development of discrete trial training (DTT) procedure in smart applied behavior analysis (Smart ABA) for autism. J Psychol Behav Stud.

[21] Nohelty K, Bradford CB, Hirschfeld L, Miyake CJ, Novack MN (2022). Effectiveness of telehealth direct therapy for individuals with autism spectrum disorder. Behav Anal Pract.

[22] Sigafoos J, Carnett A, O'Reilly M, Lancioni G (2019). Discrete trial training: a structured learning approach for children with ASD. Behavioral Interventions in Schools: Evidence-Based Positive Strategies.

[23] Lerman D, Valentino A, LeBlanc L (2016). Discrete trial training. Early Intervention for Young Children With Autism Spectrum Disorder.

[24] Hamdan M (2018). Developing a proposed training program based on discrete trial training (DTT) to improve the non-verbal communication skills in children with autism spectrum disorder (ASD). Int J Spec Educ.

[25] Eikeseth S, Smith D, Klintwall L (2014). Discrete trial teaching and discrimination training. Handbook of Early Intervention for Autism Spectrum Disorders.

[26] Faul F, Erdfelder E, Lang AG, Buchner A (2007). G*Power 3: a flexible statistical power analysis program for the social, behavioral, and biomedical sciences. Behav Res Methods.

[27] (2023). IBM SPSS Statistics GradPack and Faculty Packs. https://www.ibm.com/products/spss-statistics/gradpack.

[28] (2024). World Medical Association: Declaration of Helsinki. https://www.wma.net/policies-post/wma-declaration-of-helsinki-ethical-principles-for-medical-research.

