# Correction: Impacts of Hyperbaric Oxygen Therapy (HBOT) on Verbal Scores in Children With Autism: A Secondary Analysis of the HBOT Trial Using Multivariate Analysis of Variance (MANOVA)

**DOI:** 10.7759/cureus.c245

**Published:** 2025-08-13

**Authors:** Tami Peterson, Jessica Dodson, Sheila Burgin, Robert Sherwin, Frederick Strale,

**Affiliations:** 1 Hyperbaric Oxygen Therapy, The Oxford Center, Brighton, USA; 2 Applied Behavior Analysis, The Oxford Center, Brighton, USA; 3 Hyperbaric Oxygen Therapy, Wayne State University School of Medicine, Detroit, USA; 4 Biostatistics, The Oxford Center, Brighton, USA

The Conflicts of Interest statement has been updated to declare the following:

Financial relationships: Tami Peterson, Jessica Dodson, Sheila Burgin, Robert Sherwin and Frederick Strale Jr. declare employment from The Oxford Center. 

